# Case Report: Severe hypertriglyceridaemia and multivessel coronary artery disease – management and plaque characteristics

**DOI:** 10.3389/fcvm.2025.1622667

**Published:** 2025-12-08

**Authors:** Baiba Kokina, Maris Lapsovs, Rudolfs Roze, Baiba Lace, Andrejs Erglis, Karlis Trusinskis

**Affiliations:** 1 Department of Residency, Riga Stradins University, Riga, Latvia; 2 Latvian Centre of Cardiology, Pauls Stradins Clinical University Hospital, Riga, Latvia; 3 Faculty of Medicine and Life Sciences, University of Latvia, Riga, Latvia; 4 Department of Genetics and Rare Diseases, Riga East University Hospital, Riga, Latvia

**Keywords:** case report, severe hypertriglyceridaemia, premature atherosclerosis, multivessel disease, intravascular imaging, near-infrared spectroscopy, plasmapheresis

## Abstract

**Background:**

Elevated triglycerides have been established as a cardiovascular risk marker and the literature suggests an association with lipid-rich plaques. We report a case of severe hypertriglyceridaemia that did not result in lipid-rich atherosclerotic lesions.

**Case summary:**

Coronary angiography of a 54-year-old man with a triglyceride level >113.00 mmol/L revealed severe multivessel disease. Near-infrared spectroscopy (NIRS) demonstrated a low plaque lipid content, including the maximum lipid-core burden index within 4 mm of 0 in the right coronary artery (RCA), with >90% stenosis in the middle segment. To achieve a rapid reduction in the triglyceride level, intravenous administration of insulin and heparin combined with subsequent plasmapheresis was used, and a triglyceride level of 5.79 mmol/L was achieved before discharge. Genetic testing confirmed familial hypertriglyceridaemia with a pathogenic variant in the *lipoprotein lipase* gene.

**Conclusions:**

In a patient with severely elevated serum triglycerides and premature three-artery disease, low plaque lipid content was established with the NIRS investigation. Pharmacological management of very severe hypertriglyceridaemia with intravenous insulin and heparin therapy can rapidly decrease triglyceride levels.

## 1 Introduction

Hypertriglyceridaemia is associated with an increased risk of pancreatitis, as well as coronary artery disease (1, 2) and promotion of early atherosclerosis progression (3). Data suggest that elevated triglycerides are markers of residual cardiovascular risk and biomarkers of triglyceride-rich lipoproteins that are associated with early atherogenesis (4). Higher triglyceride levels have been associated with vulnerable plaque characteristics in intravascular imaging including lipid-rich lesions (5).

We present a case that demonstrates intravascular imaging results and management of coronary artery disease on the background of severely elevated serum triglycerides. Diagnostic and therapeutic approaches for extreme hypertriglyceridaemia are also addressed.

## 2 Case description

### 2.1 Patient presentation

A 54-year-old man was admitted for a scheduled coronary angiography with complaints of pressing and stabbing pain on the left side of the chest that was not associated with physical exercise. Patients height was 187 cm, weight – 100 kg with the corresponding body mass index (BMI) of 28.6 kg/m^2^. Outpatient clinic blood test results before hospitalization revealed a triglyceride level of 96.24 mmol/L. The blood test results are summarized in Table 1.

**Table 1:** Ambulatory blood test results.

Parameter	Value	Reference range
Red blood cells, ×10^^12^/L	5.70	4.50–5.90
Haemoglobin, g/L	200.00	131.00–175.00
Haematocrit, %	48.00	40.00–51.00
White blood cells, ×10^9^/L	4.36	4.00–9.80
Thrombocytes, ×10^9^/L	169.00	150.00–410.00
Alanine transaminase (ALT), U/L	16.00	<41.00
Aspartate transaminase (AST), U/L	13.00	<50.00
Creatinine, µmol/L	64.00	30.00–106.00
Potassium, mmol/L	3.45	3.50–5.30
Glucose, mmol/L	11.78	4.11–5.89
HbA1c, %	8.08	<6.00
Total cholesterol, mmol/L	12.99	<5.00
High-density lipoprotein cholesterol (HDL-C), mmol/L	0.67	≥1.00
Low-density lipoprotein cholesterol (LDL-C), mmol/L	0.81	<3.00
Triglycerides, mmol/L	96.24	<1.70
Brain natriuretic peptide (BNP), pg/mL	9.08	<32.80

### 2.2 Past medical history

**Figure 1:**
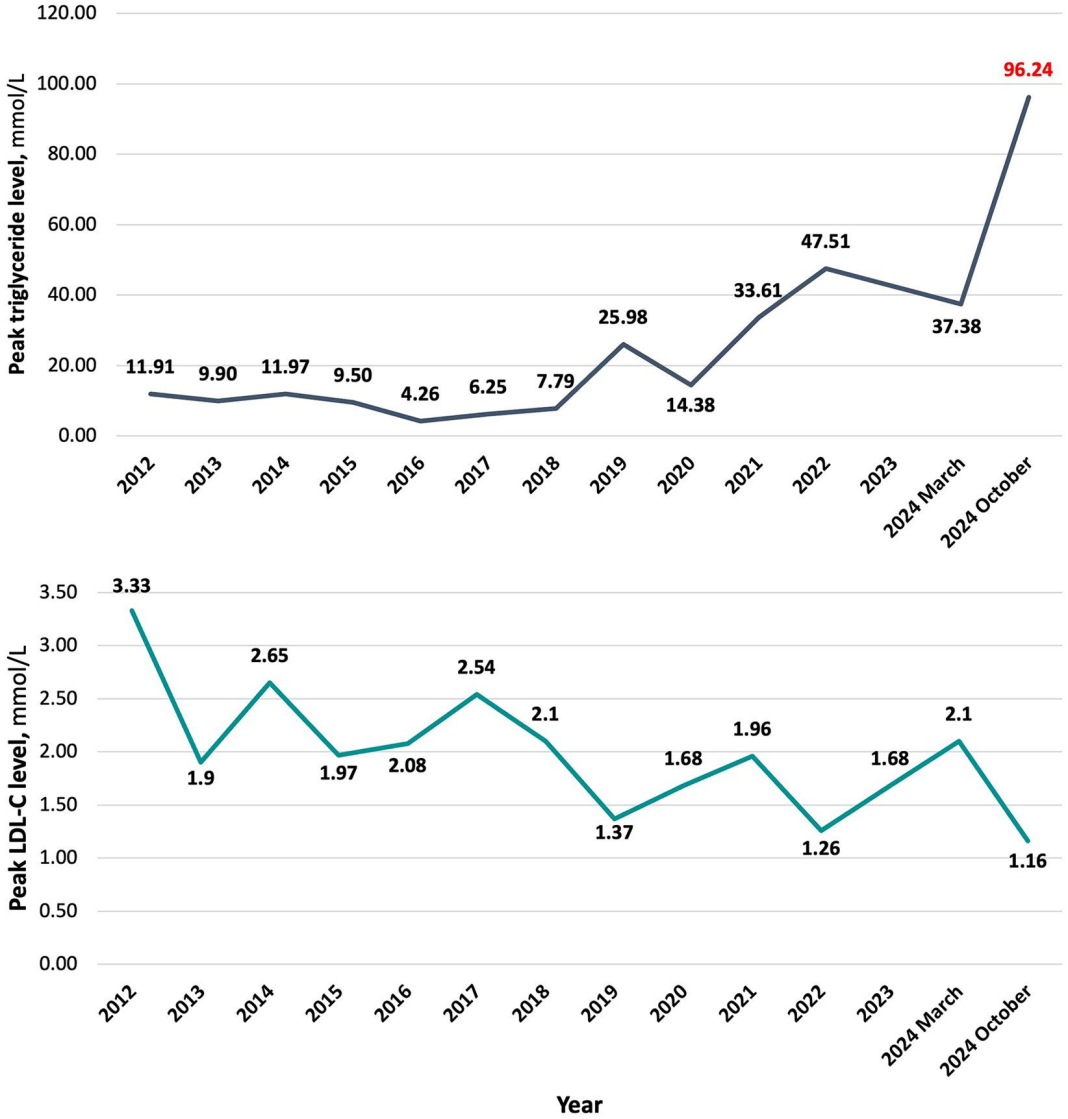
Peak triglyceride and LDL-C level dynamics.

The patient had a known history of type 2 diabetes for approximately 10 years and dyslipidaemia. Three years ago, he had been hospitalized with an acute pancreatitis episode. Triglyceride and low-density lipoprotein cholesterol (LDL-C) peak level dynamics since 2012 are summarized in Figure 1. All LDL-C values were determined with direct measurement. At the time of diagnosis of diabetes, HbA1c level was 6.70%, subsequently levels ranging around 6.50%–8.00% on the background of treatment. Peak HbA1c level throughout all years of having the diagnosis was 9.19%, detected approximately 6 months before the admission. The medications taken on a regular basis were atorvastatin 80 mg o.d., empagliflozin/metformin 12.5/1,000 mg b.i.d., gliclazide 60 mg o.d., and weekly injections of 1 mg semaglutide. Regarding lipid-lowering therapy – he had been taking atorvastatin for around 6 months. Previously, 40 mg of rosuvastatin were used for approximately a year. He had been a current smoker with a history of approximately 30 pack years and admitted to alcohol consumption once every two weeks in amount of around four to five units. There was no data on family history of premature atherosclerotic cardiovascular events or death (before age 55 in men and 60 in women).

### 2.3 Investigations and diagnostics

**Figure 2:**
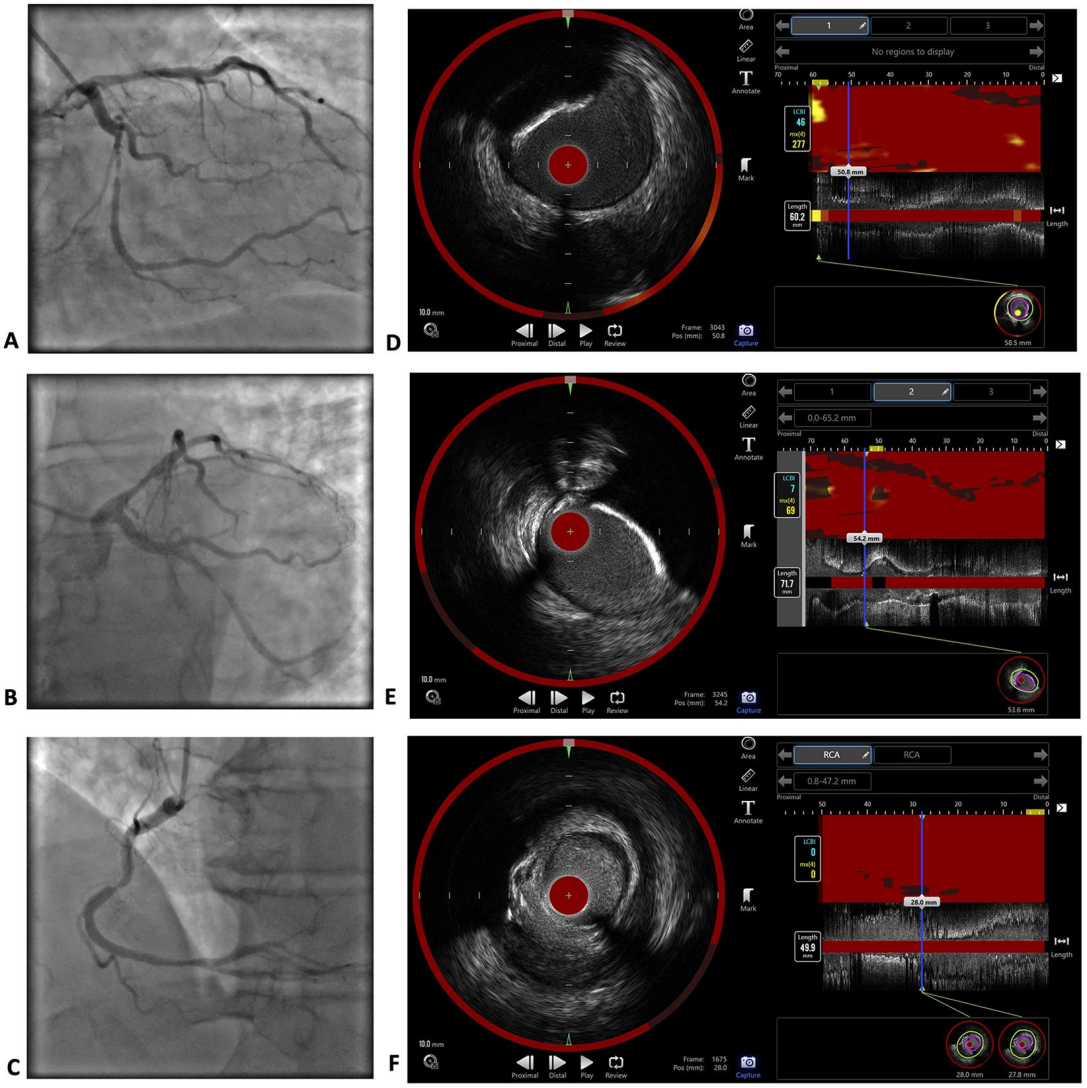
Coronary angiography and NIRS-IVUS imaging results. Coronary angiography images of the LM, LAD and LCx **(A,B)** and RCA **(C)** The investigation revealed critical stenoses >90% in the middle third of the LCx and middle third of the RCA, as well as 60%–70% borderline stenoses in the proximal LAD and distal part of the RCA. NIRS-IVUS images of the LAD **(D)**, LCx **(E)** and RCA **(F)** The maxLCBI4 mm in the RCA was 0, which corresponds to no lipid content. The maxLCBI4 mm was 277 in the LAD, and 69 in the LCx.

Coronary angiography revealed three-vessel disease with critical >90% stenoses of LCx middle segment and RCA middle third. Additionally, near-infrared spectroscopy-intravascular ultrasound (NIRS-IVUS) was performed for the RCA, LCx and LAD using an automated Dualpro™ catheter (Infraredx, Inc., Burlington, MA, USA) with a 0.5 mm/s pullback. The pullback was performed from the distal third of a coronary artery to the ostium (LM was included in the LAD and LCx pullbacks). The NIRS investigation of the RCA revealed a maximum lipid-core burden index within 4 mm (maxLCBI4 mm) of 0, which corresponds to no lipid content. MaxLCBI4 mm in the LCx and LAD were 69 and 277, respectively; nevertheless, the largest lipid content in the LAD was proximal to the most significant part of the lesion. Coronary angiography and NIRS-IVUS images are presented in Figure 2.

Given the presence of significant coronary artery disease, dopplerography of the brachiocephalic and leg arteries was performed, in which only initial atherosclerosis was detected. To evaluate changes in the pancreas with severe hypertriglyceridaemia, abdominal ultrasound was performed, and no signs of pathology were detected.

Blood tests in the hospital revealed triglyceride levels >113.00 mmol/L. Additionally, lipoprotein(a) testing was performed, with a value of 2.3 nmol/L. The high-sensitivity c-reactive protein (hs-CRP) concentration was 1.02 mg/L. To differentiate elevated haemoglobin level, the erythropoietin level was tested, and a normal value of 18.80 µIU/mL was detected. Lipase level of 59 U/L also did not indicate pathology.

The differential diagnosis for hypertriglyceridaemia included familial chylomicronaemia syndrome due to extreme triglyceride levels, familial hypertriglyceridaemia and combined hypertriglyceridaemia. Secondary hypertriglyceridaemia was also considered due to risk factors such as diabetes mellitus and alcohol consumption. Nevertheless, with such severe elevation of triglyceride levels, secondary hypertriglyceridaemia was unlikely to be the only explanation. Genetic testing revealed a heterozygous pathogenic variant in the *lipoprotein lipase* (*LPL*) gene NM_000237.3(LPL):c.701C>T (p.Pro234Leu). This genetic change is well known, and in the heterozygous state is considered pathogenic, confirming the diagnosis of familial hypertriglyceridaemia (ORPHA:444490). Patients family testing was also performed, and this pathology was not found in children.

### 2.4 Management

Percutaneous coronary intervention with two drug-eluting stents (DESs) was performed for the RCA first, followed by scheduled angioplasty for the LCx with a DES and the LAD with a DES.

Since patient had extremely high triglyceride levels with risk for pancreatitis, alongside necessity to perform prompt percutaneous coronary intervention with DES implantation under these circumstances with limited data in this regard, there was a need for rapid triglyceride level reduction. Standard pharmacotherapy including fibrates was not expected to demonstrate results that quickly, as well as insufficient effect for such high triglycerides was foreseen. Therefore, to manage severely elevated triglycerides, it was decided to perform plasmapheresis. The first procedure was unsuccessful because of filter clogging due to the large amount of triglycerides and chylous blood. Therefore, there was a need to reduce triglyceride levels via pharmacotherapy. Data regarding rapid triglyceride level lowering is mostly based on published case reports and case series. Insulin and heparin are suggested for quick and effective triglyceride reduction (6). Insulin therapy dosing along with intravenous fluid therapy was calculated and administered following case series-based recommendations published by Hoff and Piechowski (7). Intravenous insulin was administered at a rate of 10 units/hour for patient's weight 100 kg. To avoid hypoglycaemia, 250 mL of 5% glucose solution every hour was used every hour. To prevent hypokalaemia, 20 mEq of potassium chloride premix in 250 mL of Ringer's solution was also administered in parallel every hour. Additionally, intravenous heparin with a bolus dose of 4,000 IU, followed by 1,000 IU/hour infusion in normal saline premix, was used. Dosing of heparin was done according to the standard scheme used in our hospital. After two hours of intensive intravenous insulin and heparin therapy, a triglyceride level of 71.17 mmol/L was achieved. Afterwards, successful plasmapheresis could be performed, and three procedures (two consecutive days and skipping one day) were performed. Before plasmapheresis sessions, intravenous insulin treatment was repeated according to the described scheme. At the time of discharge, the triglyceride level was 5.79 mmol/L. Triglyceride level dynamics while in the hospital are summarized in Table 2.

**Table 2:** Triglyceride level dynamics in the hospital.

Parameter	Admission	After intravenous insulin and heparin therapy	After first plasmapheresis session	After second plasmapheresis session	After third plasmapheresis session
Triglycerides, mmol/L	>113.00	71.17	30.50	25.26	5.79

Strict lifestyle modifications were discussed with the patient: a fat-free diet, no added sugars, and alcohol abstinence were recommended.

For long-term hypolipidaemic therapy, atorvastatin/ezetimibe 40/10 mg o.d., fenofibrate 200 mg o.d. and icosapent ethyl 2 g b.i.d. were prescribed. Statin and ezetimibe combination, as well as fibrate were started on the first day of admission, while patient started taking icosapent ethyl only after a couple of months due to limited availability of medication in our country. Taking into consideration high risk of pancreatitis, semaglutide therapy for diabetes was discontinued as a potential additional contributing factor (8, 9).

### 2.5 Follow-up and outcomes

After two weeks, the triglyceride level of the patient increased to 50.26 mmol/L. From the prescribed lipid-lowering therapy, the patient had not started taking icosapent ethyl but was adherent to the intake of other medications. The lack of icosapent ethyl in therapy is explained by the limited availability of medication in the country. The patient was not strictly compliant with lifestyle modifications, nevertheless understanding the importance of this component of treatment. After discussion with the patient, in addition to pharmacological treatment, plasmapheresis sessions were continued in an outpatient setting. With this treatment, triglyceride levels were maintained at approximately 12 mmol/L. Both pharmacotherapy and plasmapheresis sessions are continued, with good tolerability and no adverse events.

## 3 Discussion

A patient with extreme hypertriglyceridaemia and severe coronary artery disease is presented.

Elevated triglyceride levels have been associated with atherosclerotic cardiovascular disease (10) and increased cardiovascular event risk, including the mechanism of low-grade inflammation (11, 12). *Post-hoc* analysis of TNT study has demonstrated an association of elevated triglyceride-rich lipoprotein (TRL) levels and adverse cardiovascular event risk (13), triglycerides being the remnant markers of TRL catabolism (14). A recently published study by Shuitema et al. has shown the relation of elevated triglyceride levels to residual cardiovascular risk in patients with established cardiovascular disease, leading to higher cardiovascular events and mortality, irrespective of other lipid target achievement and intensity of lipid-lowering therapy (15). Other supporting data have been published in a PESA study subgroup analysis including patients with low to moderate cardiovascular risk. In this group even in patients having normal LDL-C levels, elevated triglyceride levels were associated with subclinical atherosclerosis and vascular inflammation (16). In the presented patient an increased level of triglycerides has been present for many years and furthermore, demonstrating progressively negative dynamics. Therefore, based on available data, triglycerides should be considered as an important contributor to the extensive coronary artery disease in our patient. Nevertheless, other cardiovascular risk factors are also important, including, smoking and long history of diabetes with insufficient metabolic compensation. Interestingly, in our patient, a low lipid content in the coronary artery lesions was detected with NIRS. In the RCA with critical stenosis, the maxLCBI4 mm value was 0, which corresponds to no lipid content. A possible explanation could be provided by the fact that triglyceride particles that are carried in TRLs – chylomicrons and very-low-density lipoproteins – can not cross the endothelium due to their size (4). While LDL-C is known to be a causal factor for atherosclerotic cardiovascular disease, triglycerides are thought to have greater contributions through metabolic dysregulation and proinflammatory effects on endothelial cells and macrophages (2, 17), potentially promoting the early onset of atherosclerosis. TRLs with high content of triglycerides have demonstrated upregulation of tumor necrosis factor-alpha (TNF-alpha), thus inducing expression of vascular cell adhesion molecule-1 (VCAM-1) in endothelial cells, leading to monocyte adhesion (18). Nevertheless, it is important to emphasize that remnant particles produced through TRL lipolysis are sufficiently small to penetrate subendothelial space and be taken up by arterial wall macrophages (19). Additionally, these remnants are even more atherogenic than LDL-C (20). Another important consideration is investigations performed on high-dose statin therapy and good LDL-C control. The YELLOW trial demonstrated plaque stabilization by lipid burden reduction as evaluated by NIRS in patients taking high-intensity rosuvastatin (21). The EASY-FIT study involving optical coherence tomography revealed a greater increase in plaque fibrous cap thickness and lipid arc reduction with 20 mg atorvastatin therapy than with 5 mg atorvastatin therapy (22). Our patient had been on high-intensity statin treatment for around 1.5 years – 1 year of 40 mg of rosuvastatin and 6 months of 80 mg of atorvastatin, which therefore could have contributed to plaque delipidation at the moment of intravascular imaging being performed.

Familial hypertriglyceridaemia affects approximately 1% of the population, and triglyceride levels usually reach 11.2 mmol/L (23). Regarding the genetic basis, in our patient a heterozygous mutation in *LPL* gene was found. Mutations in *LPL* gene, but when in autosomal recessive biallelic pathogenic variant or compound heterozygous variant, cause more than 80% of cases of familial chylomicronemia syndrome that is an extremely rare variant of hypertriglyceridaemia (prevalence is in the range of 1:100 000 to 1:1 000 000) with a very prominent effect. In familial hypertriglyceridaemia the origin is typically either polygenic with cumulative effect of multiple common variants with smaller effect on triglyceride levels, or rare heterozygous variants with a larger effect on triglyceride levels. One of the genes with heterozygous variant implicated in familial hypertriglyceridaemia is the *LPL* gene, affecting the function of LPL (24). LPL variants are the most prevalent pathogenic variants in heterozygous patients, accounting for 60%–80% of cases (25). In a cohort of patients with severe hypertriglyceridaemia in Brazil, the most frequent variant in *LPL* gene was the c.701C>T (p.Pro234Leu) (26), which was also the one detected in our patient. In patients with severe hypertriglyceridaemia in Italy, this was also one of the mutations detected (27). Overall allele frequency is the highest in Europe (7:100 000), it is absent from African and Asia populations (28), but has a strong founder effect in French-Canadian population (29). Heterozygous patients have residual lipolytic capacity of LPL, however secondary factors can overwhelm already genetically compromised lipolysis (25). Our patient had a combination of genetically determined hypertriglyceridaemia with type 2 diabetes, alcohol consumption, and smoking.

The state of hypertriglyceridaemia can interfere with haematological laboratory values. In our patient high haemoglobin level alongside normal red blood cell count and haematocrit was detected. For differentiation of the cause, erythropoietin level was determined, which was normal. Literature data suggests false increase of haemoglobin in samples with high triglycerides with the common colorimethric method, since triglycerides increase blood turbidity (30). Regarding LDL-C estimation, elevated triglycerides do not have significant impact on the values with the use of direct enzymatic assays, while the mathematical Friedewald formula can not be used in cases of severe hypertriglyceridaemia (31). LDL-C values estimated in our patient were all detected by direct assays. Interference of severely elevated triglycerides with NIRS imaging readouts has not been described.

The American College of Cardiology Consensus document states that in patients with elevated triglyceride levels ≥5.65 mmol/L, the primary goal for lowering triglyceride levels is a reduction in pancreatitis risk. Lifestyle recommendations are one of the main hypertriglyceridaemia management pillars (32). For triglyceride-lowering pharmacotherapy with regard to cardiovascular risk reduction, the European Society of Cardiology Guidelines for the management of dyslipidaemias recommend statins as the first choice, followed by combination with icosapent ethyl and fibrate in high-risk patients (31). This strategy was recommended for our patient; nevertheless, the real-life availability of icosapent ethyl in our country attenuated the initiation of the medication. However, considering the severely elevated triglyceride levels, high pancreatitis risk, three-vessel coronary artery disease, and PCI with DES implantation in our patient, the need for rapid triglyceride level reduction was addressed. Although pharmacologial triglyceride-lowering therapy was optimized already on admission, the onset of action as well as expected extent of triglyceride reduction was not sufficient. For fibrates, the estimated triglyceride level reduction ranges are around 30%–50% (33). REDUCE-IT trial demonstrated tiglyceride lowering by 18.3% baseline to 1 year (34). In addition, omega-3 unsaturated fatty acids, specifically icosapent ethyl, are recomenndef with focus on cardiovascular event reduction (35). Therefore, plasmapheresis was the method of choice; nevertheless, the first session was unsuccessful because the plasma separator was clogged with chylous plasma, thus the initial triglyceride level had to be lowered with pharmacological methods. Case series data suggest the efficacy and safety of intravenous insulin and heparin administration for rapid triglyceride lowering. Insulin has the capacity to promote LPL synthesis and activation, whereas heparin helps dissociate heparan sulfate from LPL, resulting in the acceleration of lipoprotein metabolism (7, 36). In our case, dosing calculations were performed based on previously published data, and effective triglyceride level reduction from >113.00 mmol/L to 5.79 mmol/L was achieved by combining a pharmacological approach and plasmapheresis. However, at the time of follow-up, the maintenance of the results was not permanent, presumably due to a lack of compliance with lifestyle modifications.

## 4 Conclusions

In a patient with severe hypertriglyceridaemia, premature three-artery disease with low plaque lipid content was established. Effective triglyceride level reduction was achieved by combining pharmacological treatment and plasmapheresis.

## Data Availability

The raw data supporting the conclusions of this article will be made available by the authors, without undue reservation.
